# Structure and inference in annotated networks

**DOI:** 10.1038/ncomms11863

**Published:** 2016-06-16

**Authors:** M. E. J. Newman, Aaron Clauset

**Affiliations:** 1 Department of Physics, University of Michigan, 450 Church Street, Ann Arbor, Michigan 48109, USA; 2 Center for the Study of Complex Systems, University of Michigan, 450 Church Street, Ann Arbor, Michigan 48109, USA; 3 Santa Fe Institute, 1399 Hyde Park Road, Santa Fe, New Mexico 87501, USA; 4 Department of Computer Science, University of Colorado, 430 UCB, Boulder, Colorado 80309, USA; 5 BioFrontiers Institute, University of Colorado, 596 UCB, Boulder, Colorado 80309, USA

## Abstract

For many networks of scientific interest we know both the connections of the network and information about the network nodes, such as the age or gender of individuals in a social network. Here we demonstrate how this ‘metadata' can be used to improve our understanding of network structure. We focus in particular on the problem of community detection in networks and develop a mathematically principled approach that combines a network and its metadata to detect communities more accurately than can be done with either alone. Crucially, the method does not assume that the metadata are correlated with the communities we are trying to find. Instead, the method learns whether a correlation exists and correctly uses or ignores the metadata depending on whether they contain useful information. We demonstrate our method on synthetic networks with known structure and on real-world networks, large and small, drawn from social, biological and technological domains.

## Abstract (web summary)

Analysis of network structure is usually based on knowledge of connections alone, ignoring additional information such as gender or age of individuals in social networks. Here the authors devise an approach that incorporates such metadata and uses it to improve the detection of network communities.

## Graphical abstract

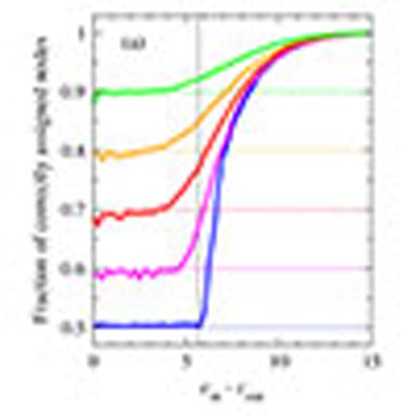

Networks arise in many fields and provide a powerful and compact representation of the internal structure of a wide range of complex systems^1^. Examples include social networks of interactions among people, technological and information networks such as the Internet or the World Wide Web, and biological networks of molecules, cells, or entire species. The last two decades have witnessed rapid growth both in the availability of network data and in the number and sophistication of network analysis techniques. Borrowing ideas from graph theory, statistical physics, computer science, statistics and other areas, network analysis typically aims to characterize a network's structural features in a way that sheds light on the behaviour of the system the network describes. Studies of social networks, for instance, might identify the most influential or central individuals in a population. Studies of road networks can shed light on traffic flows or bottlenecks within a city or country. Studies of pathways in metabolic networks can lead to a more complete understanding of the molecular machinery of the cell.

Most research in this area treats networks as objects of pure topology, unadorned sets of nodes and their interactions. Most network data, however, are accompanied by annotations or metadata that describe properties of nodes such as a person's age, gender or ethnicity in a social network, feeding mode or body mass of species in a food web, data capacity or location of nodes on the Internet and so forth. (There can be metadata on the edges of a network as well as on the nodes^2^, but our focus here is on the node case.) In this paper, we consider how to extend the analysis of networks to directly incorporate such metadata. Our approach is based on methods of statistical inference and can in principle be applied to a range of different network analysis tasks. Here we focus specifically on one of the most widely studied tasks, the community detection problem. Community detection, also called node clustering or classification, searches for a good division of a network's nodes into groups or classes^3^. Typically, one searches for assortative structure, groupings of nodes such that connections are denser within groups than between them. This structure is common in social networks, for example, where groups might correspond to sets of friends or co-workers, but it also occurs in other cases, including biological and ecological networks, the Web, transportation and distribution networks, and others. Less common, but no less important, is disassortative structure, in which network connections are sparser within groups than between them, and mixtures of assortative and disassortative structure can also occur, where different groups may have varying propensities for within- or between-group connections.

In some cases, the groups identified by community detection correlate meaningfully with other network properties or functions, such as allegiances or personal interests in social networks^3^,^4^ or biological function in metabolic networks^5^,^6^. Some recent research, however, has suggested that these cases may be the exception rather than the rule^7^,^8^, an important point that we address later in this paper.

A large number of methods have been proposed for detecting communities in unannotated networks^3^. Among these, some of the most powerful, both in terms of rigorously provable performance and of raw speed, are those based on statistical inference. Here we build on these methods to incorporate node metadata—either categorical or real-valued—into the community detection problem in a principled and flexible manner. (For real-valued metadata we restrict ourselves to the scalar or one-dimensional case, but multi-dimensional metadata, such as locations in physical or latent space^9^,^10^,^11^, would be a natural focus for future extensions of our approach.) The resulting methods have several attractive features. First, they can make use of metadata in arbitrary format to improve the accuracy of community detection. Second, and crucially for our goals, they do not assume *a priori* that the metadata correlate with the communities we seek to find. Instead, they detect and quantify the relationship between metadata and community, if one exists, then exploit that relationship to improve the results. Even if the correlation is imperfect or noisy, the method can still use what information is present to return improved results. Conversely, if no correlation exists the method will automatically ignore the metadata, returning results based on network structure alone.

Third, our methods allow us to select between competing divisions of a network. Many networks have a number of different possible divisions^12^. For example, a social network of acquaintances may have meaningful divisions along lines of age, gender, race, religion, language, politics or many other variables. By incorporating metadata that correlate with a particular division of interest, we can favour that division over others, steering the analysis in a desired direction. (Approaches like this are sometimes referred to as supervised learning techniques, particularly in the statistics and machine-learning literature.) Thus, if we are interested for instance in a division of a social network along lines of age, and we have age data for some fraction of the nodes, we can use those data to steer the algorithm towards age-correlated divisions. Even if the metadata are incomplete or noisy, the algorithm can still use them to guide its analysis. However, if we hand the algorithm metadata that do not correlate with any good division of the network, the method will decline to follow along blindly, and will inform us that no good correlation exists.

Finally, the correlation between metadata and network structure learned by the algorithm (if one exists) is interesting in its own right. Once found, it allows us to quantify the agreement between network communities and metadata, and to predict community membership for nodes for which we lack network data and have only metadata. If we have learned, for example, that age is a good predictor of social groupings, then we can make quantitative predictions of group membership for individuals about whom we know their age and nothing else.

A number of other researchers have investigated ways to incorporate metadata into community detection calculations^13^,^14^,^15^,^16^,^17^,^18^,^19^, though they have typically made stronger assumptions about the nature of the communities or metadata, assuming, for example, that communities are always assortative, or that the metadata represent locations in physical space. Perhaps closest to our approach are semi-supervised learning methods^17^,^20^,^21^,^22^, where it is assumed that we are given the exact community assignments of some fraction of the nodes and the goal is to deduce the reminder. A variant of this approach is active learning, in which the community membership of some nodes is given, but the known nodes are not specified *a priori*, being instead chosen by the algorithm itself as it runs^23^,^24^. Another vein of research, somewhat further from our approach, considers the case where we are told some pairs of nodes that are either definitely in or definitely not in the same community, and then assigns communities subject to these constraints^25^,^26^.

Our approach, which is described in detail in the Methods section, takes as input a network accompanied by a set of node metadata, which may be, for instance, numerical values or arbitrary textual or alphanumeric labels, and produces as output a division of the nodes of the network into a specified number *k* of groups or communities. The method does not (as some methods do) assume a particular pattern of connections among communities—such as denser connections within groups than between them—and it is numerically efficient, making use of a so-called belief propagation scheme to perform rapid inference of the optimal group assignments making possible applications to very large networks. The largest network we have analysed using the method has over 1.4 million nodes.

In the following sections we give results showing that our method is able to recover known communities in benchmark data sets with higher accuracy than algorithms based on network structure alone, that we can select between competing community divisions in both real and synthetic tests, that the method is able accurately to divine correlations between network structure and metadata, or determine that no such correlation exists, and that learned correlations between structure and metadata can be used to predict community membership based on metadata alone.

## Results

### Synthetic networks

Our first tests are on computer-generated (synthetic) networks that have known community structure embedded within them. These networks were created using the stochastic block model, a standard model of network structure in which *n* nodes are assigned to groups then edges are placed between them independently with probabilities that are a function of group membership only^27^,^28^. After the networks are created, we generate discrete-valued node metadata that match the true community assignments of nodes a given fraction of the time, and are chosen randomly from the non-matching values otherwise. This allows us to control the extent to which the metadata correlate with the community structure and hence test the algorithm's ability to make use of metadata of varying quality.

**Figure 1:**
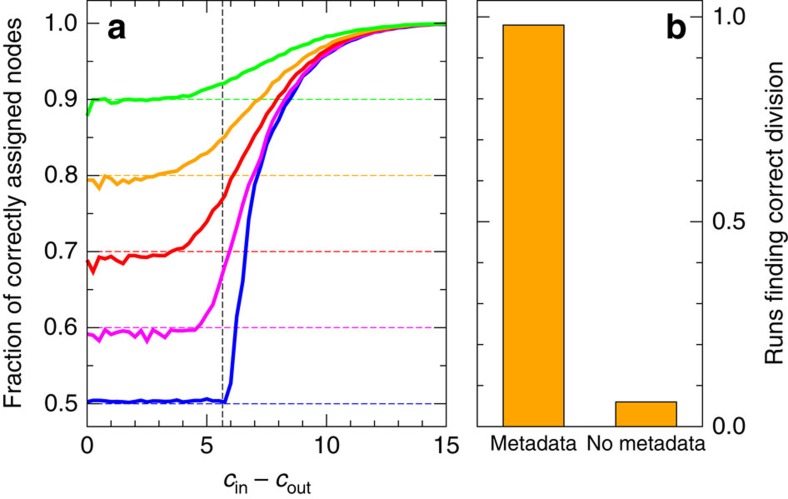
Tests on synthetic benchmark networks with *n*=10,000 nodes. (**a**) Fraction of correctly assigned nodes for networks with two planted communities with mean degree *c*=8, as a function of the difference between the numbers of within- and between-group connections. The five curves show results for networks with a match between metadata and planted communities on a fraction 0.5, 0.6, 0.7, 0.8 and 0.9 of nodes (bottom to top). The vertical dashed line indicates the theoretical detectability threshold, below which no algorithm without metadata can detect the communities. (**b**) Fraction of 100 four-group test networks where the algorithm selects a particular two-way division, out of several competing possibilities, with and without the help of metadata that are weakly correlated with the desired division. A run is considered to find the correct division if the fraction of correctly classified nodes exceeds 85%. Network parameters are *c*_out_=4 and *c*_in_=20.

Figure 1a shows results for a set of such networks having two communities of equal size, with edge probabilities *p*_in_=*c*_in_/*n* and *p*_out_=*c*_out_/*n* for within-group and between-group edges, respectively, where *n* is the number of nodes as before and *c*_in_ and *c*_out_ are constants whose values we choose. When *c*_in_ is much greater than *c*_out_ the communities are easy to detect from network structure alone, but as *c*_in_ approaches *c*_out_ the structure becomes weaker and harder to detect. Each curve in the figure shows the fraction of nodes classified into their correct groups by our algorithm, as we vary the strength of the community structure, measured by the difference *c*_in_−*c*_out_. Individual curves show results for different levels of correlation between communities and metadata.

When metadata and community agree for exactly half of the nodes (bottom curve) there is no correlation between the two, and the metadata cannot help in community detection. It thus comes as no surprise that this curve shows the lowest success rate. At higher levels of correlation the metadata contain useful information and the algorithm's performance improves accordingly.

Examining the figure, a clear pattern emerges. For large *c*_in_−*c*_out_ the network contains strong community structure and the algorithm reliably classifies essentially all nodes into the correct groups, as we would expect of any effective algorithm. As the structure weakens the fraction of correct nodes declines, but it remains higher in all the cases where the metadata are useful than in the lowest curve where they are not. Moreover, the algorithm's success rate appears to improve monotonically with the level of correlation between metadata and communities.

When there are no metadata, it is known that the belief propagation algorithm we use gives optimal answers to the community detection problem in the sense that no other algorithm will classify a higher fraction of nodes correctly on average^29^. The fact that our algorithm does better when there are metadata thus implies that the algorithm with metadata does better than any possible algorithm without metadata.

Furthermore, it has previously been shown that below the so-called detectability threshold, which occurs at 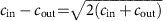 (indicated by the vertical dashed line in the figure, and aligning with the sharp transition in the bottom curve), community structure becomes so weak as to be undetectable by any algorithm that relies on network structure alone^29^,^30^. Well below this threshold, however, our algorithm still correctly classifies a fraction of the nodes roughly equal to the fraction of metadata that match the communities, meaning that the algorithm does better with metadata than without it even below the threshold. Figure 1a also shows that the fraction of correctly classified nodes beats this baseline level for values of *c*_in_−*c*_out_ somewhat below the threshold, suggesting that the use of the metadata shifts the threshold downward or perhaps eliminates it altogether.

In short, our method automatically combines the available information from network structure and metadata to do a better job of community detection than any algorithm based on network structure alone. And when either the network or the metadata contain no information about community structure the algorithm correctly ignores them and returns an estimate based only on the other.

Figure 1b shows a different synthetic test, of the algorithm's ability to select between competing divisions of a network. In this test, networks were generated with four equally sized communities but the algorithm was tasked with finding a division into just two communities. There are eight ways of dividing such a network in two if we are to keep the four underlying groups undivided. We imagine a situation in which we are interested in finding a particular one out of these eight. A conventional community detection algorithm might find a reasonable division of these networks, but there is no guarantee it would find the ‘correct' one—some fraction of the time we can expect it to find one of the competing divisions. But if our algorithm is given a set of metadata that correlate with the division of interest, even if the correlation is poor, then that division will be favoured over the others.

In our tests the desired division was one that places two of the underlying four groups in one community and the remaining two in the other. Two-valued metadata were generated that agree with this division 65% of the time, a relatively weak level of correlation, not far above the 50% of completely uncorrelated data. Nonetheless, as shown in Fig. 1b, this is enough for the algorithm to reliably find the correct division of the network in almost every case—98% of the time in our tests. Without the metadata, by contrast, we succeed only 6% of the time. Some practical applications of this ability to select among competing divisions are given in the next section.

### Real-world networks

In this section we describe applications of our method to a range of real-world networks, drawn from social, biological and technological domains.

For our first application we analyse a network of school students, drawn from the US National Longitudinal Study of Adolescent Health. The network represents patterns of friendship, established by survey, among the 795 students in a medium-sized American high school (US grades 9–12, ages 14–18 years) and its feeder middle school (grades 7 and 8, ages 12–14 years).

Given that this network combines middle and high schools, it comes as no surprise that there is a clear division (previously documented) into two network communities corresponding roughly to the two schools. Previous work, however, has also shown the presence of divisions by ethnicity^31^. Our method allows us to select between divisions by using metadata that correlate with the one we are interested in.

**Figure 2:**
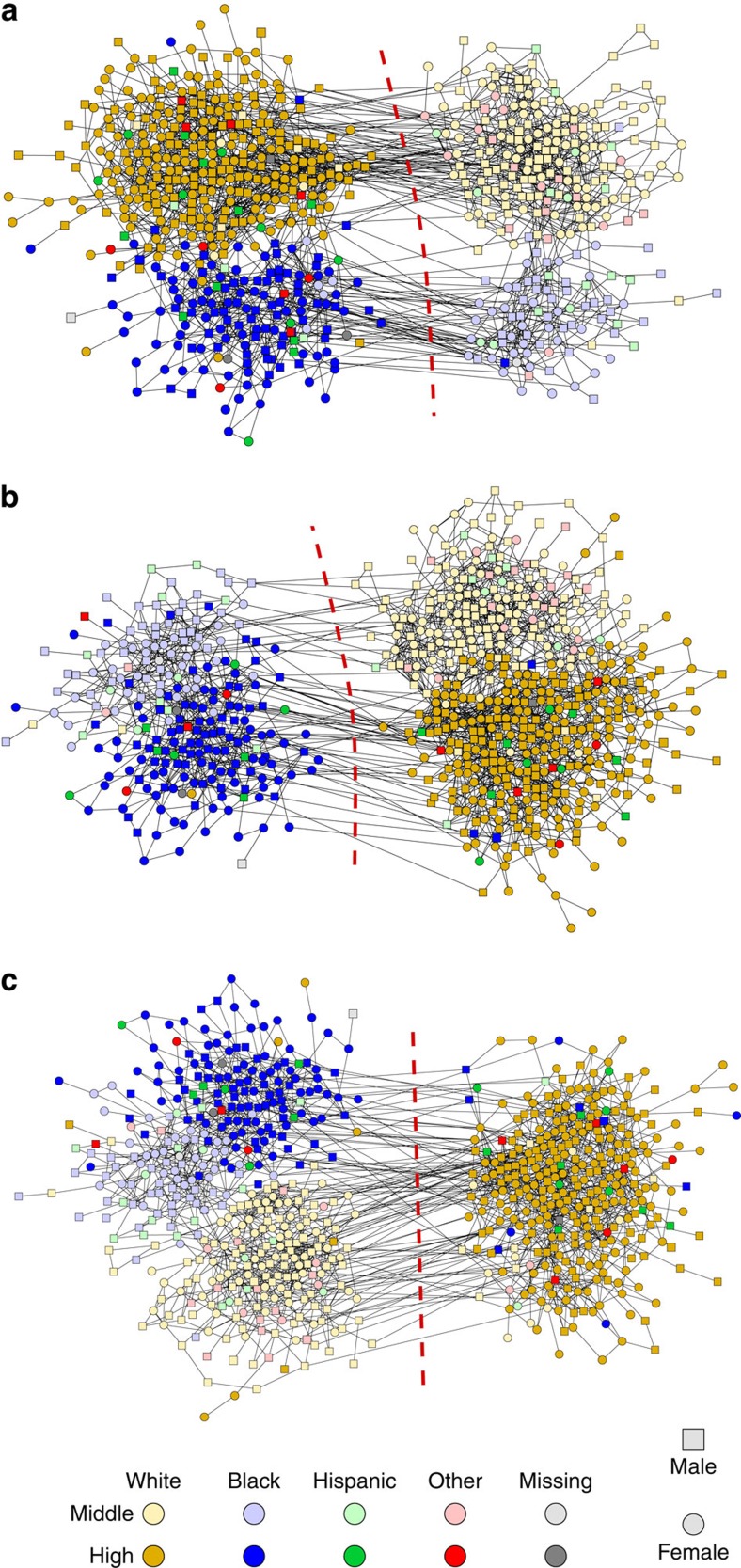
Communities found in a high school friendship network with various types of metadata. Three divisions of a school friendship network, using as metadata (**a**) school grade, (**b**) ethnicity and (**c**) gender.

Figure 2 shows the results of applying our algorithm to the network three times. Each time, we asked the algorithm to divide the network into two communities. In Fig. 2a, we used the six school grades as metadata and the algorithm readily identifies a division into grades 7 and 8 on the one hand and grades 9–12 on the other—that is, the division into middle school and high school. In Fig. 2b, by contrast, we used the students' self-identified ethnicity as metadata, which in this data set takes one of four values: white, black, hispanic, or other (plus a small number of nodes with missing data). Now the algorithm finds a completely different division into two groups, one group consisting principally of black students and one of white. (The small number of remaining students are distributed roughly evenly between the groups.)

One might be concerned that in these examples the algorithm is mainly following the metadata to determine community membership, and ignoring the network structure. To test for this possibility, we performed a third analysis, using gender as metadata. When we do this, as shown in Fig. 2c, the algorithm does not find a division into male and female groups. Instead, it finds a new division that is a hybrid of the grade and ethnicity divisions (white high-school students in one group and everyone else in the other). That is, the algorithm has ignored the gender metadata, because there was no good network division that correlated with it, and instead found a division based on the network structure alone. The algorithm makes use of the metadata only when doing so improves the quality of the network division (in the sense of the maximum-likelihood fit described in the Methods section).

The extent to which the communities found by our algorithm match the metadata (or any other ‘ground truth' variable) can be quantified by calculating a normalized mutual information (NMI)^32^,^33^, as described in the Methods section. NMI ranges in value from 0 when the metadata are uninformative about the communities to 1 when the metadata specify the communities completely. The divisions shown in Fig. 2a,b have NMI scores of 0.881 and 0.820, respectively, indicating that the metadata are strongly though not perfectly correlated with community membership. By contrast, the division in Fig. 2c, where gender was used as metadata, has an NMI score of 0.003, indicating that the metadata contain essentially zero information about the communities.

Our next application is to an ecological network, a food web of predator–prey interactions between 488 marine species living in the Weddell Sea, a large bay off the coast of Antarctica^34^,^35^. A number of different metadata are available for these species, including feeding mode (deposit feeder, suspension feeder, scavenger and so on), zone within the ocean (benthic, pelagic and so on) and others. In our analysis, however, we focus on one in particular, the average adult body mass. Body masses of species in this ecosystem have a wide range, from microorganisms weighing nanograms or less to hundreds of tonnes for the largest whales. Conventionally, in such cases one often works with the logarithm of mass, which makes the range more manageable, and we do so here. Then we perform *k*-way community decompositions using this log-mass as metadata, for various values of *k*.

**Figure 3:**
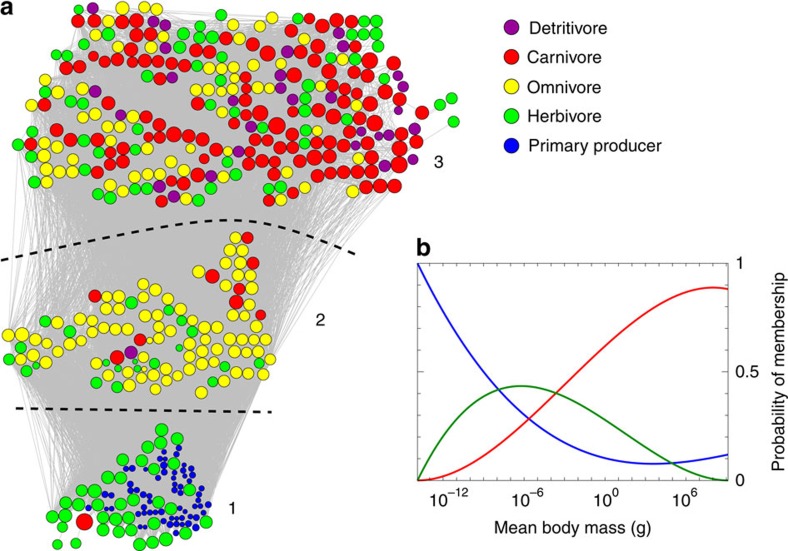
Results of the application of the method of this paper to the food web of marine species in the Weddell Sea. (**a**) Three-way decomposition of the marine food web described in the text, with the logarithm of mean body mass used as metadata. Node sizes are proportional to log-mass, and colours indicate species role within the ecosystem. (**b**) Learned probabilities of belonging to each of the communities as a function of body mass. We use log mass as the metadata variable in our calculations, but the horizontal axis here is calibrated to read in terms of the original mass in grams using a logarithmic scale. The blue, green and red curves correspond, respectively, to the communities labelled 1, 2 and 3 in **a**.

Figure 3a shows the results for *k*=3. Nodes are coloured according to their role in the ecosystem—carnivores, herbivores, primary producers and so forth. The division found by the algorithm appears to match these roles quite closely, with one group composed almost entirely of primary producers and herbivores, one of omnivores and a third that contains most of the carnivores. Node sizes in the figure are proportional to log-mass, which increases as we go up the figure, indicating that the algorithm has recovered from the network structure the well-known correlation between body mass and ecosystem role^36^. This point is further emphasized by the probabilities of membership in the three groups, which are an incidental, but often useful, additional output of the algorithm we use (see Methods). These probabilities, plotted as a function of body mass in Fig. 3b, show that low-mass organisms are overwhelmingly likely to be in the first group, and high-mass ones in the third group. Organisms of intermediate mass have a broader distribution, but are particularly concentrated in the second group.

The membership probabilities are also of interest in their own right. If, for instance, we were to learn of a new species, previously unrepresented in our food-web data set, then even without knowing its pattern of network connections we can make a statement about its probability of belonging to each of the communities, as well as its probability of interaction with other species, so long as we know its body mass. For instance, a low body mass of 10^−12^ g would put a species with high probability in group 1 in Fig. 3, meaning it is almost certainly a primary producer or a herbivore, with the interaction patterns that implies.

Community detection is widely studied precisely because network communities are believed to be correlated with network function. More specifically, it is commonly assumed that communities correlate with some underlying functional variable, which may or may not be observed. This assumption, however, has been challenged by recent work that compared communities in real-world networks against ‘ground truth' metadata variables and found little correlation between the two^7^,^8^. This is a striking discovery, but there is a caveat. As we have seen, there are often multiple meaningful community divisions of a network (as in the school friendship network of Fig. 2, for example), and the fact that one division is uncorrelated with a given metadata variable does not rule out the possibility that another could be.

Our third real-world example application illustrates these issues using one of the same networks studied in ref. 8, a 46,676-node representation of the peering structure of the Internet at the level of autonomous systems. The ‘ground truth' variable for this network is the country in which each autonomous system is located. The analysis of ref. 8 found there to be little correlation between community structure and countries.

We first analyse this network without metadata, performing a traditional ‘blind' community division, into five groups using standard methods. We then repeat the analysis using the algorithm of this paper, with the countries as metadata. Recall that, in doing this, we do not force the algorithm to find a community division that aligns with the metadata if no such division exists, but if a division does exist it will be favoured over competing divisions that do not align with the metadata. There are 173 distinct countries in the data set, a significantly larger number of metadata values than for any of the other networks we have considered, but by no means beyond the capabilities of our method.

As before, we assess the results using the normalized mutual information. If indeed there are many competing divisions of the network, only some of which correlate with the particular metadata we are given, then we would expect our blind analysis to return a range of NMI values on different runs, some low and (maybe) some higher. This is indeed what we see, with the NMI in our calculations ranging from a high of 0.626 to a relatively low 0.398, the latter being in agreement with results quoted in ref. 8. Conversely, when the algorithm of this paper is applied with countries as metadata, we find an NMI score significantly higher than any of these figures, at 0.870, which would conventionally be interpreted as an indication of strong correlation.

These results emphasize that an apparent lack of correlation between network communities and metadata could be the result of the presence of competing network divisions, which are not correlated with the particular metadata we have at hand. The algorithm of this paper allows us to select among divisions and hence find ones that correlate with the variable of interest.

Our fourth example is drawn from the FB100 data set of Traud *et al.*^37^, which is a set of friendship networks among college students at the US universities compiled from friend relations on the social networking website Facebook. The networks date from the early days of Facebook when its services were available only to universities and each university formed a separate and unconnected subgraph in the larger network. The nodes in these networks represent the participants, who are mainly though not exclusively students, the edges represent friend relations on Facebook, and in addition to the network structure there are metadata of several types, including gender, college year (that is, year of college graduation), major (that is, principal subject of students' study, if known) and a numerical code indicating which dorm students lived in.

The primary divisions in these networks appear to be by age, or more specifically by college year. For instance, we have looked in some detail at the network for Harvard University, the birthplace of Facebook, which has 15,126 nodes. Most of these represent undergraduate students, who span college years 2003–2009, but there are also a small number of alumni (that is, former students), primarily those recently graduated (graduation years 2000–2002), as well as grad students, summer students, and some faculty and staff.

**Figure 4:**
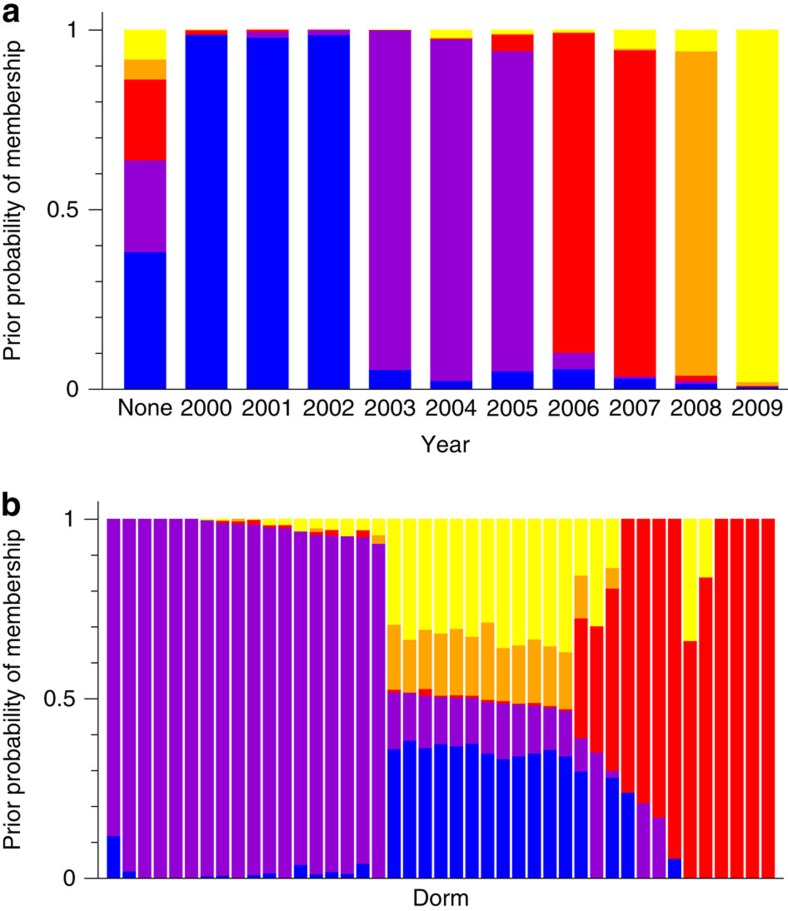
Learned prior probability of community membership for two five-way divisions of the Harvard Facebook friendship network described in the text. The horizontal axis is (**a**) year of graduation and (**b**) dormitory, and the colours represent the learned prior probabilities of membership in each of the communities.

Figure 4a shows results from a five-way division of the network using our algorithm with year as metadata. This calculation provides another example of the usefulness of the learned probabilities of group membership in shedding light on the structure of the network. The figure shows a visualization of the probabilities as a function of year, with the colours showing the relative probability of belonging to each of the communities. Each of the bars in the plot has the same height of 1 since the probabilities are required to sum to 1, while the balance of colours shows the distribution over communities. Examination of the top panel in the figure shows clearly a division of the network along age lines. Two groups, in orange and yellow at the right of the plot, correspond to the most recent two years of students at the time of the study (graduation years 2008 and 2009) and the next, in red, account for the two years before that (2006 and 2007). The purple community corresponds to the next three years, 2003–2005, while the sixth group, shown in blue, corresponds to the alumni. Finally, students for whom year was not recorded are shown in the column marked ‘None,' which is a mixture of all five groups.

These results align well with the original analysis of the same data by Traud *et al.*^37^, who performed a traditional community division of the network and then carried out *post hoc* statistical tests to measure correlations between communities and metadata. They found strong correlations with college year metadata, in agreement with our results. With the benefit of hindsight the results may appear unsurprising—anyone who has been to college knows that a large number of your friends are in the same year as you—but one could certainly formulate competing hypotheses. One alternative that Traud *et al.* considered was that friendship might be influenced by where students live, with students living in the same dormitory more likely to be friends, regardless of what year they are in. Traud *et al.* found that there was some evidence for this hypothesis, but that the effect was weaker than that for age, and our analysis confirms this. The bottom panel in Fig. 4 shows a plot of the priors for a division with dorm as the metadata variable and there is a clear correlation between dorm and community membership, but it is not as clean as in the case of age. There appear to be two groups that align strongly with particular sets of dorms (coloured red and purple in the figure) while the rest of the dorms are a mix of different communities (the region in the middle of the figure). The impression that the community structure is more closely aligned with graduation year than with dormitory is also borne out by the normalized mutual information values for the two divisions, which are 0.668 for graduation year but 0.255 for dormitory.

Our final real-world network example is drawn from a gene recombination network for the human parasite *Plasmodium falciparum*, which causes malaria. Malaria is endemic in tropical regions and is responsible for roughly a million deaths annually, mostly children in sub-Saharan Africa^38^. During infection, parasites evade the host immune system and prolong the infection by repeatedly changing a protein camouflage displayed on the surface of an infected red blood cell. To enable this behaviour, each parasite has a repertoire of roughly 60 immunologically distinct proteins, each of which is encoded by a *var* gene in the parasite's genome^39^. These genes undergo frequent recombination, producing novel proteins by shuffling and splicing substrings from existing *var* genes.

The process of recombination induces a natural bipartite network with two types of nodes, *var* genes on the one hand and their constituent substrings on the other, where each gene node is connected by an edge to every substring it contains^40^,^41^. Recombination in these genes occurs mainly within a number of distinct highly variable regions (HVRs) and each HVR represents a distinct set of edges among the same nodes. Here we focus on the one-mode gene–gene projections of the HVR 5 and HVR 6 subnetworks, which have previously been analysed using community detection methods without metadata^40^,^41^. Each of these one-mode networks consists of 297 genes.

We analyse these networks using as metadata the Cys labels derived from the HVR 6 sequence and the Cys-PoLV (CP) labels derived from the sequences adjacent to HVRs 5 and 6 (refs 39, 42, 43). Both types of labels depend only on the sequences' characteristics: Cys indicates the number of cysteines the HVR 6 sequence contains (2 or 4) while CP subdivides the Cys classifications into six groups depending on particular sequence motifs. Thus, each node has two metadata values, a Cys label and a CP label. The Cys labels are biologically important because cysteine counts have been implicated in severe disease phenotypes^39^,^42^.

**Figure 5:**
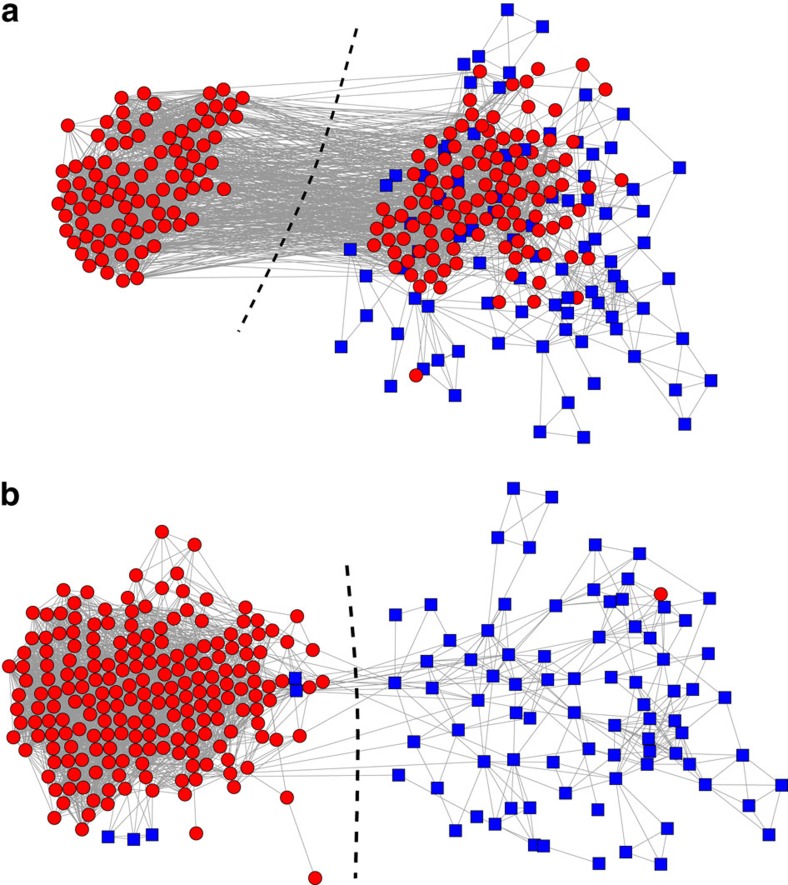
Inferred communities for the malaria HVR 6 gene recombination network. Communities inferred (**a**) without metadata and (**b**) with metadata for the HVR 6 network of the human malaria parasite *P. falciparum*, where metadata values are the CP labels for the genes and nodes are coloured according to their biologically relevant Cys label.

In our calculations we use the six CP labels as metadata for a two-way community division of the network and then evaluate the degree to which the inferred communities correlate with the Cys metadata. Figure 5 shows the results for the HVR 6 network with and without the CP labels as metadata. Without metadata, the Cys labels are mixed across the inferred groups (Fig. 5a), but with metadata we obtain a nearly perfect partition (Fig. 5b). This indicates that the CP label correlates well with the network's community structure, a fact that was obscured in the analysis without metadata. Furthermore, the inferred communities correlate strongly with the coarser Cys labels, which were not shown to the method: observing that a gene has two cysteines is highly predictive (96% probability) of that gene being in one group, while having four cysteines is modestly predictive (67% probability) of being in the other group. Thus, the method has discovered by itself that the motif sequences that define the CP labels, along with their corresponding network communities, correlate with cysteine counts and their associated severe disease phenotypes^39^,^42^.

**Figure 6:**
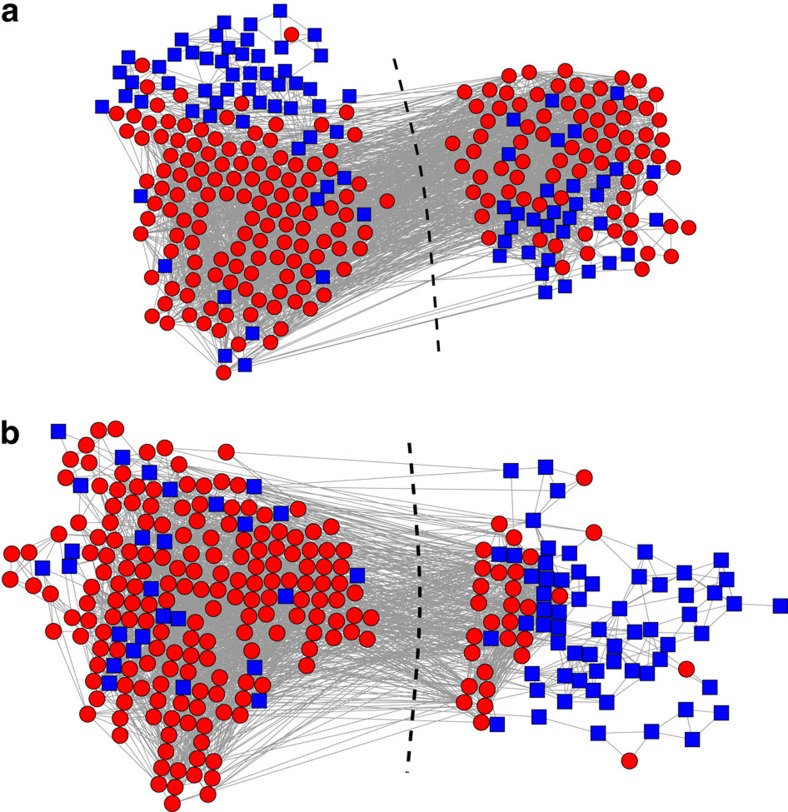
Inferred communities for the malaria HVR 5 gene recombination network. Communities inferred (**a**) without metadata and (**b**) with metadata for the HVR 6 network of the human malaria parasite *P. falciparum*, where metadata values are the CP labels for the genes and nodes are coloured according to their biologically relevant Cys label.

The communities in the HVR 6 network represent highly non-random patterns of recombination, which are thought to indicate functional constraints on protein structure. Previous work has conjectured that common constraints on recombination span distinct HVRs^40^. We can test this hypothesis using the methods described in this paper. There is no reason *a priori* to expect that the community structure of HVR 6 should correlate with that of HVR 5 because the Cys and CP labels are derived from outside the HVR 5 sequences—Cys labels reflect cysteine counts in HVR 6 while CP labels subdivide Cys labels based on sequence motifs adjacent to, but outside of, HVR 5. Applying our methods to HVR 5 without any metadata (Fig. 6a), we find mixing of the HVR 6 Cys labels across the HVR 5 communities. By contrast, using the CP labels as metadata for the HVR 5 network, our method finds a much cleaner partition (Fig. 6b), indicating that indeed the HVR 6 Cys labels correlate with the community structure of HVR 5.

## Discussion

There are a number of possible extensions of this work. At the simplest level one could include more complex metadata types, such as combinations of discrete and continuous variables, or vector variables such as spatial coordinates. Metadata could also be incorporated into methods for detecting other types of structure, such as hierarchies^44^, motifs^45^, core-periphery structures^46^, rankings^47^ or latent-space structures^48^. And the resulting fits could form the starting point for a variety of additional applications, such as the prediction of missing links or missing metadata in incomplete data sets. These and other possibilities we leave for future work.

## Methods

Our method makes use of techniques of Bayesian statistical inference in which we construct a generative network model possessing the specific features we hope to find in our data, namely community structure and a correlation between that structure and node metadata, then we fit the model to an observed network plus accompanying metadata and the parameters of the fit tell us about the structure of the network.

The model we use is a modified version of a stochastic block model. The original stochastic block model, proposed in 1983 by Holland *et al.*^27^, is a simple model for generating random networks with community structure in which nodes are divided among some number of communities and edges are placed randomly and independently between them with probabilities that depend only on the communities to which the nodes belong. We modify this model in two ways. First, following ref. 28, we note that the standard stochastic block model does poorly at mimicking the structure of networks with highly heterogeneous degree sequences (which includes nearly all real-world networks), and so we include a ‘degree-correction' term that matches node degrees (that is, the number of connections each node has) to those of the observed data. Second, we introduce a dependence on node metadata via a set of prior probabilities. The prior probability of a node belonging to a particular community becomes a function of the metadata, and it is this function that is learned by our algorithm to incorporate the metadata into the calculation.

### Unordered data

Consider an undirected network with *n* nodes labelled by integers *u*=1 … *n*, divided among *k* communities, and denote the community to which node *u* belongs by *s*_*u*_∈1 … *k*. In the simplest case, we consider metadata with a finite number *K* of discrete, unordered values and we denote node *u*'s metadata by *x*_*u*_∈1 … *K*. The choice of labels 1 … *K* is arbitrary and does not imply an ordering for the metadata or that the metadata are one-dimensional. If a social network has two-dimensional metadata describing both language and race, for example, we simply encode each possible language/race combination as a different value of *x*: English/white, Spanish/white, English/black and so forth. If a network has nodes that are missing metadata values, we just let ‘missing' be another metadata value.

Given metadata **x**={*x*_*u*_} and degree **d**={*d*_*u*_} for all nodes, a network is generated from the model as follows. First, each node *u* is assigned to a community *s* with a probability depending on *u*'s metadata *x*_*u*_. The probability of assignment we denote *γ*_*sx*_ for each combination *s*,*x* of community and metadata, so the full prior probability on community assignments is 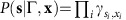, where **Γ** denotes the *k* × *K* matrix of parameters *γ*_*sx*_. (More complex forms of the prior are appropriate in other cases, as we will see.) Once every node has been assigned to a community, edges are placed independently at random between nodes, with the probability of an edge between nodes *u* and *v* being


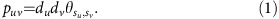


where *θ*_*st*_ are parameters that we specify, with *θ*_*st*_=*θ*_*ts*_. The factor *d*_*u*_*d*_*v*_ allows the model to fit arbitrary degree sequences as described above. Models of this kind have been found to fit community structure in real networks well^28^.

Community detection then consists of fitting the model to observed network data using the method of maximum likelihood. Given an observed network, we define its adjacency matrix **A** to be the *n* × *n* real symmetric matrix with elements *a*_*uv*_=1, if there is an edge between nodes *u* and *v* and 0 otherwise. Then the probability, or likelihood, that this network was generated by our model, given the parameters and metadata, is


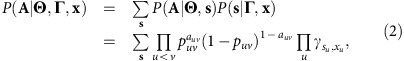


where **Θ** is the *k* × *k* matrix with elements *θ*_*st*_ and the sum is over all possible community assignments **s**.

Fitting the model involves maximizing this likelihood with respect to **Θ** and **Γ** to determine the most likely values of the parameters, which we do using an expectation-maximization (EM) algorithm. Typically, rather than maximizing (2) itself, we maximize instead its logarithm,


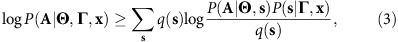


which gives the same answer for **Θ** and **Γ** but is often more convenient. The most obvious approach for performing the maximization would be simply to differentiate with respect to the parameters, set the result to zero, and solve the resulting equations. This, however, produces a complex set of implicit equations that have no easy solution. Instead, therefore, we make use of Jensen's inequality, which says that for any set of positive quantities *x*_*i*_ the log of their sum obeys


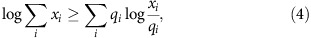


where *q*_*i*_ is any correctly normalized probability distribution such that ∑_*i*_*q*_*i*_=1. Note that the exact equality is recovered by the particular choice


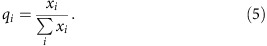


Applying Jensen's inequality to equation (3), we find that


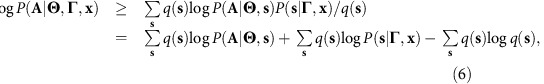


where *q*(**s**) is any distribution over community assignments **s** such that ∑_**s**_*q*(**s**)=1. The maximum of the right-hand side of this inequality with respect to possible choices of the distribution *q*(**s**) coincides with the exact equality, which, following equation (5), is when


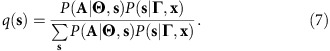


Thus, the maximization of the left-hand side of (6) with respect to **Θ**, **Γ** to give the optimal values of the parameters is equivalent to a maximization of the right-hand side both with respect to *q*(**s**) (which makes it equal to the left-hand side) and with respect to **Θ**, **Γ**. A simple algorithm for performing such a double maximization is to repeatedly maximize with respect to first *q*(**s**) and then **Θ**, **Γ** until we converge to an answer. In other words:

1. Make an initial guess about the parameter values and use them to calculate the optimal *q*(**s**) from equation (7).
2. Using that value, maximize the right-hand side of (6) with respect to the parameters, while holding *q*(**s**) constant.
3. Repeat from step 1 until convergence is achieved.

Step 2 can be performed by differentiating with *q*(**s**) fixed and subject to the normalization constraint ∑_*s*_*γ*_*sx*_=1 for all *x*. Performing the derivatives and assuming that the network is large and sparse so that *p*_*uv*_ is small, we find to leading order in small quantities that


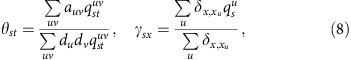


where


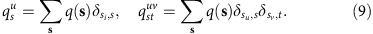


In addition, for a large sparse network, the community assignments of distant nodes will be uncorrelated and hence we can write 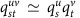 in the denominator of (8) to get


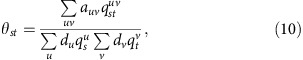


which reduces the denominator sums from *n*^2^ terms to only *n* and considerably speeds the calculation. (We cannot make the same factorization in the numerator, since the terms in the numerator involve 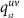 on adjacent nodes *u*, *v* only, so the nodes are not distant from one another.)

Equation (7) tells us that once the iteration converges, the value of *q*(**s**) is


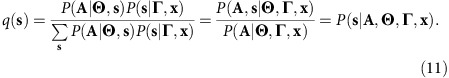


In other words *q*(**s**) is the posterior distribution over community assignments **s**, the probability of an assignment **s** given the inputs **A**, **Θ**, **Γ**, and **x**, and 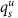 is the marginal posterior probability that node *u* belongs to community *s*. Normally, in fact, 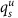 is the object of primary interest in the calculation, as it tells us to which group each node belongs. That is, it tells us the optimal division of the network into communities. As discussed in the Results section, the prior probabilities *γ*_*sx*_ may also be of interest, since they tell us how and to what extent the metadata are correlated with the communities. If the metadata are uncorrelated with the network communities, the prior probabilities become constant, independent of the metadata, and thus have no impact on the posterior probabilities of the communities. Similarly, if the network is large and has strong community structure (as in the region on the right of Fig. 1a where *c*_in_−*c*_out_ is large), the prior probabilities will have little effect on the results and the algorithm will find the structure embedded in network with or without help from the metadata.

Computationally, the most demanding part of the EM algorithm is calculating the sum in the denominator of equation (7), which has an exponentially large number of terms, making its direct evaluation intractable on all but the smallest of networks. Traditionally one gets around this problem by approximating the full distribution *q*(**s**) by Monte Carlo importance sampling. In our calculations, however, we instead use a recently proposed alternative method based on belief propagation^29^, which is significantly faster, and fast enough in practice for applications to very large networks.

### Final likelihood value

The EM algorithm always converges to a maximum of the likelihood but is not guaranteed to converge to the global maximum—it is possible for there to be one or more local maxima as well. To get around this problem we normally run the algorithm repeatedly with different random initial guesses for the parameters and from the results choose the one that finds the highest likelihood value. In the calculations presented in this paper we did at least 10 such ‘random restarts' for each network. To determine which run has the highest final value of the likelihood we calculate the log-likelihood from the right-hand side of (6) using *P*(**A**|**Θ**, **s**) and *P*(**s**|**Γ**, **x**) as in equation (2), the final fitted values of the parameters **Θ** and **Γ** from the EM algorithm, and *q*(**s**) as in equation (7). (As we have said, the right-hand side of (6) becomes equal to the left, and hence equal to the true log-likelihood, when *q*(**s**) is given the value in equation (7).)

Putting it all together, our expression for the log-likelihood is


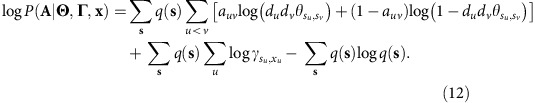


Neglecting terms beyond first order in small quantities, the first sum can be rewritten as


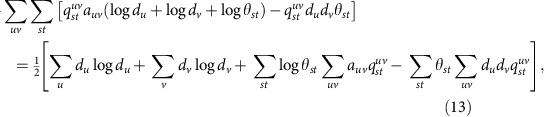


where we have made use of 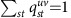 and 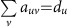.

The first two terms in (13) are constant for any given network and hence can be neglected—they are irrelevant for comparing the likelihood values between different runs on the same network. The final term can be rewritten using equation (8) as


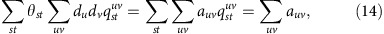


which is also a constant and can be neglected. Thus, only the third term in (13) need be carried over.

The second sum in (12) is


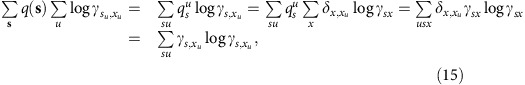


where we have used equation (8) again in the third equality.

The final sum in (12) is the entropy of the posterior distribution *q*(**s**), which is harder to calculate because it requires not just the marginals of *q* but the entire distribution. We get around this by making the so-called Bethe approximation^49^:


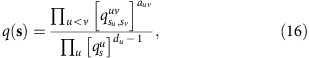


which is exact on trees and locally tree-like networks, and is considered to be a good working approximation on other networks. Substituting this form into the entropy term gives


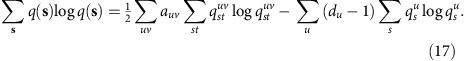


Finally, combining equations (13)–(17), 22, 23, 24, 25 and substituting into equation (12), our complete expression for the log-likelihood, neglecting constants, is


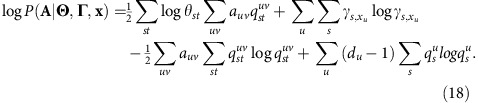


The run that returns the largest value of this quantity is the run with the highest likelihood and hence the best fit to the model.

### Ordered metadata

We also consider cases in which the metadata are ordered and potentially continuous variables, such as age or income in a social network, which require a different algorithm. The prior probability *P*(*s*|*x*) of belonging to community *s* given metadata value *x* now becomes a continuous function of *x*. In most cases the metadata have a finite range and for convenience we normalize them to fall in the range *x*∈[0, 1]. (In the rarer case of metadata with infinite range a transformation can be applied first to bring them into a finite range.) One immediate question that arises is what limitations should be placed on the form of the probability *P*(*s*|*x*). We cannot allow it to take any functional form, such as ones that vary arbitrarily rapidly, for (at least) two reasons. First, it would be unphysical—there are good reasons in most cases to believe that nodes with infinitesimally different metadata *x* have only infinitesimally different probabilities of falling in a particular group. In other words, *P*(*s*|*x*) should be smooth and slowly varying in some sense. Second, a function that can vary arbitrarily rapidly can have arbitrarily many degrees of freedom, which would lead to overfitting of the model.

To avoid of these problems, we enforce a slowly varying prior by writing the function *P*(*s*|*x*) as an expansion in a finite set of suitably chosen basis functions. In our work we use the Bernstein polynomials of degree *N*:


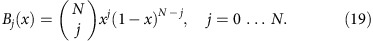


(There is an interesting model selection problem inherent in the choice of the degree, which we do not tackle here but which would be a good topic for future research.)

Bernstein polynomials have three particular properties that make them useful for representing probabilities:

1. They form a complete basis set for polynomials of degree *N*.
2. They fall in the range 0≤*B*_*j*_(*x*)≤1 for all *x*∈[0, 1] and all *j*.
3. They satisfy the sum rule

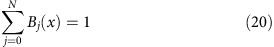


for all *x*∈[0, 1].

The first of these implies that any degree-*N* representation of the probability *P*(*s*|*x*) can be written in the form


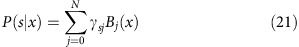


for some choice of coefficients *γ*_*sj*_. Moreover, if *γ*_*sj*_∈[0, 1] for all *s*,*j* then *P*(*s*|*x*)∈[0, 1] for all *x*∈[0, 1], meaning it is a well-defined probability within this domain. To see this observe first that *P*(*s*|*x*)≥0 when *γ*_*sj*_≥0 since all *B*_*j*_(*x*)≥0, and second that for *γ*_*sj*_≤1 we have


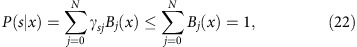


where we have made use of equation (20).

Finally, the normalization condition ∑_*s*_*P*(*s*|*x*)=1 can be satisfied for all *x* by requiring that


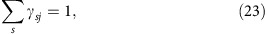


so that


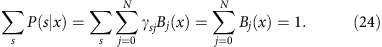


We now employ the form (21) to represent the prior probabilities in our EM algorithm, writing


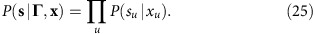


The only change to the algorithm from the previous case arises when we maximize the right-hand side of equation (6). Instead of maximizing with respect to the prior probabilities directly, we now maximize with respect to the coefficients *γ*_*sj*_ of the expansion. The optimal values of the coefficients are given by


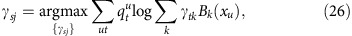


subject to the constraint (23). One can derive conditions for the maximum by direct differentiation, but the equations do not have a closed-form solution, so instead we once again employ Jensen's inequality (4) to write


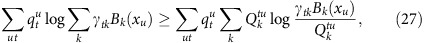


which is true for any 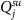 satisfying 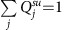 for all *u*, *s*. The exact equality is achieved when


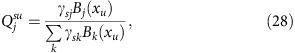


and the maximum of equation (26) can be computed by first maximizing over 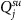 in this way and then over *γ*_*sj*_. This leads to an iterative algorithm analogous to the EM algorithm in which one computes the 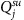 from (28) and then, using those values, computes the maximum with respect to *γ*_*sj*_ by differentiating the right-hand side of (27) subject to the condition (23), which gives


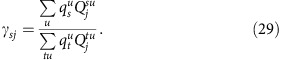


Iterating (28) and (29) alternately to convergence now gives us the coefficients *γ*_*sj*_ of the optimal degree-*N* polynomial prior. Note that (29) always gives *γ*_*sj*_ in the range from zero to one, so that, as discussed above, the resulting prior *P*(*s*|*x*) also lies between zero and one and is thus a lawful probability.

### Implementation

The calculations for this paper were implemented in the C programming language for speed. The code is included as a Supplementary Software file. We also used a number of additional techniques to improve speed and convergence. We find that the majority of the running time of the algorithm is taken up by the belief propagation calculations, and this time can be shortened by noting that highly converged values of the beliefs are pointless in early steps of the EM algorithm. The parameter values used to calculate the beliefs in these steps are, presumably, highly inaccurate since the EM algorithm has not converged yet, so there is little point waiting for the beliefs to converge to high accuracy when there are much bigger sources of error in the calculation. In the calculations of this paper, we limited the belief propagation to no more than 20 steps at any point. In the early stages of the EM algorithm this gives rather crude values for the beliefs, but these values would not be particularly good under any circumstances, no matter how many steps we used, because of the poor parameter values. In the later stages of the EM algorithm, 20 steps are enough to ensure good convergence (and indeed we often get good convergence after many fewer steps than this).

We also place a limit on the total number of iterations of the EM algorithm, discarding results that fail to converge within the allotted time. In the calculations in this paper, this second limit was set at either 20 or 100 steps. We have performed some runs with higher limits (up to 1,000 EM steps) but, paradoxically, we find this often gives poorer results, for instance in our tests on synthetic networks. This seems to be because the EM algorithm sometimes converges (as we have said) to the wrong solution and empirically when it does so it also often converges more slowly. By discarding runs that converge slowly, therefore, we tend to discard incorrect solutions and improve the average quality of our results.

### Normalized mutual information

In our calculations we make use of normalized mutual information to measure the quality of our results. NMI is a widely used measure of the level of agreement between community divisions and ‘ground truth' variables, proposed by Danon *et al.*^32^. Given a community division represented by an *n*-element vector **s** of group labels and discrete metadata represented by **x**, the conditional entropy of the community division is^50^


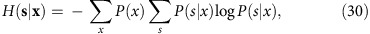


*P*(*x*) is the fraction of nodes with metadata *x* and *P*(*s*|*x*) is the probability that a node belongs to community *s* if it has metadata *x*. Traditionally the logarithm is taken in base 2, in which case the units of conditional entropy are bits. The conditional entropy is equal to the amount (in bits) of additional information one would need, on top of the metadata themselves, to specify the community membership of every node in the network. If the metadata are perfectly correlated with the communities, so that knowing the metadata tells us the community of every node, then the conditional entropy is zero. Conversely, if the metadata are worthless, telling us nothing at all about community membership, then the conditional entropy takes its maximum value, equal to the total entropy of the community assignment *H*(**s**)=−∑_*s*_*P*(*s*)log *P*(*s*). In our case we already know the value of *P*(*s*|*x*): it is equal to the prior probability *γ*_*sx*_ of belonging to community *s*, one of the outputs of our algorithm. Hence


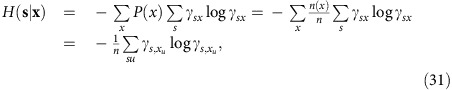


where *n*(*x*)=*nP*(*x*) is the number of nodes with metadata *x* and *n* is the total number of nodes in the network, as previously.

Alternatively, if we want a measure that increases (rather than decreases) with the amount of information the metadata give us, we can subtract *H*(**s**|**x**) from *H*(**s**), which gives the (unnormalized) mutual information


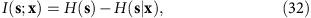


This quantity has a range from zero to *H*(**s**), making it potentially hard to interpret, so commonly one normalizes it, creating the normalized mutual information. There are several different normalizations in use. As discussed by McDaid *et al.*^33^, it is mathematically reasonable to normalize by the larger, the smaller or the mean of the entropies *H*(**s**) and *H*(**x**) of the communities and metadata. Danon *et al.*^32^ originally used the mean, while Hric *et al.*^8^ in their work on lack of correlation between communities and metadata (discussed in the Results section) used the maximum. In the present case, however, we contend that the best choice is the minimum.

The largest possible value of the mutual information is *H*(**s**), which sets the scale on which the mutual information should be considered large or small. Thus, one might imagine the correct normalization would be achieved by simply dividing *I*(**s**;**x**) by *H*(**s**), yielding a value that runs from zero to one. This, however, would give a quantity that was asymmetric with respect to **s** and **x**—if the values of the two vectors were reversed the value of the mutual information would change. Mutual information, by convention, is symmetric and we would prefer a symmetric definition. Dividing by min[*H*(**s**), *H*(**x**)] achieves this. In all the examples we consider, the number of communities is less than the number of metadata values, in some cases by a wide margin. Assuming the values of both to be reasonably broadly distributed, this implies that the entropy *H*(**s**) of the communities will be smaller than that of the metadata *H*(**x**) and hence min[*H*(**s**), *H*(**x**)]=*H*(**s**). Thus if we define


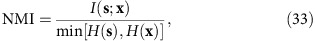


we ensure that the normalized mutual information lies between zero and one, that it has a symmetric definition with respect to **s** and **x**, and that it will achieve its maximum value of one when the metadata perfectly predict the community membership. Other definitions, normalized using the mean or maximum of the two entropies, satisfy the first two of these three conditions but not the third, giving values smaller than one by an unpredictable margin even when the metadata perfectly predict the communities. We use the definition (33) in the calculations presented in this paper.

### Data availability

Additional materials are available online. The US National Longitudinal Study of Adolescent Health data referenced in this study are available from Add Health, Carolina Population Center, 123W. Franklin Street, Chapel Hill, NC 27516-2524 (addhealth@unc.edu).

## Additional information

**How to cite this article:** Newman, M. E. J. *et al.* Structure and inference in annotated networks. *Nat. Commun.* 7:11863 doi: 10.1038/ncomms11863 (2016).

## Supplementary Material

Supplementary Software 1 An implementation, in the C programming language, of the described methods.
